# AI-based detection and classification of distal radius fractures using low-effort data labeling: evaluation of applicability and effect of training set size

**DOI:** 10.1007/s00330-021-07811-2

**Published:** 2021-03-19

**Authors:** Patrick Tobler, Joshy Cyriac, Balazs K. Kovacs, Verena Hofmann, Raphael Sexauer, Fabiano Paciolla, Bram Stieltjes, Felix Amsler, Anna Hirschmann

**Affiliations:** 1 University Hospital Basel, University of Basel, Clinic of Radiology and Nuclear Medicine, University of Basel, Petersgraben 4, 4031 Basel, Switzerland; 2 Amsler Consulting Basel, Gundeldingerrain 111, 4059 Basel, Switzerland

**Keywords:** Radiography, Radius fractures, Deep learning

## Abstract

**Objectives:**

To evaluate the performance of a deep convolutional neural network (DCNN) in detecting and classifying distal radius fractures, metal, and cast on radiographs using labels based on radiology reports. The secondary aim was to evaluate the effect of the training set size on the algorithm’s performance.

**Methods:**

A total of 15,775 frontal and lateral radiographs, corresponding radiology reports, and a ResNet18 DCNN were used. Fracture detection and classification models were developed per view and merged. Incrementally sized subsets served to evaluate effects of the training set size. Two musculoskeletal radiologists set the standard of reference on radiographs (test set A). A subset (B) was rated by three radiology residents. For a per-study-based comparison with the radiology residents, the results of the best models were merged. Statistics used were ROC and AUC, Youden’s J statistic (J), and Spearman’s correlation coefficient (ρ).

**Results:**

The models’ AUC/J on (A) for metal and cast were 0.99/0.98 and 1.0/1.0. The models’ and residents’ AUC/J on (B) were similar on fracture (0.98/0.91; 0.98/0.92) and multiple fragments (0.85/0.58; 0.91/0.70). Training set size and AUC correlated on metal (ρ = 0.740), cast (ρ = 0.722), fracture (frontal ρ = 0.947, lateral ρ = 0.946), multiple fragments (frontal ρ = 0.856), and fragment displacement (frontal ρ = 0.595).

**Conclusions:**

The models trained on a DCNN with report-based labels to detect distal radius fractures on radiographs are suitable to aid as a secondary reading tool; models for fracture classification are not ready for clinical use. Bigger training sets lead to better models in all categories except joint affection.

**Key Points:**

*• Detection of metal and cast on radiographs is excellent using AI and labels extracted from radiology reports.*

*• Automatic detection of distal radius fractures on radiographs is feasible and the performance approximates radiology residents.*

*• Automatic classification of the type of distal radius fracture varies in accuracy and is inferior for joint involvement and fragment displacement.*

## Introduction

Acute distal radius fractures are common traumatic injuries and comprise 17% of all fractures in western societies [1]. Distal radius fractures can be diagnosed confidently on wrist radiographs [2]. These radiographs are often seen by non-specialized physicians. If radiographs and clinical symptoms are ambiguous to the emergency or family doctor, an inaccurate diagnosis or treatment delay may occur. Automated fracture detection and reporting may reduce diagnostic uncertainty and aid in flagging radiographs for referral to a specialist and support the workflow by providing a preliminary radiology report. The number of publications using deep learning (DL), a computationally demanding subcategory of artificial intelligence (AI), has steeply increased in recent years. The rapid evolvement of DL has only been possible due to widely available graphic processor units that meet the needs of DL. A category of DL is known as deep convolutional neural networks (DCNN), which addresses the underlying architecture. DCNNs are well suited for pattern detection on images. They have successfully been used for fracture detection and localization on radiographs [3–12]. Training data for automated fracture detection have been heterogeneously labeled by orthopedic surgeons [5], orthopedic specialists [6], radiology [10, 11, 13, 14] or orthopedic [15] residents and general radiologists [4] or specialized musculoskeletal radiologists [7, 8]. Cheng et al [8] used registry data to label hip fractures on radiographs and only Olczak et al [12] used key phrases of radiology reports to label radiographs for the training set. While the potential of laborious expert-based data labeling is well described, the potential of labels extracted by a key phrase search is unclear. To date, five studies have evaluated the automated detection of distal radius fractures on radiographs with promising sensitivities and specificities of 81–98% and 73–100%, respectively [4–6, 12, 13]. In order to generate a useful radiology report, an algorithm for fracture classification beyond fracture detection and localization is required. Moreover, the ideal number of radiographs to train and test an algorithm for peripheral fracture detection is unclear and studies utilized varying numbers ranging from 524 to 65,264 radiographs [12, 13].

The main purpose of this study was to evaluate the performance of a DCNN in detecting and classifying distal radius fractures, metal, and cast on wrist radiographs using labels based on unstructured radiology reports. The secondary aim was to evaluate the effect of the training set size on the algorithm’s performance.

## Materials and methods

Institutional review board approval was waived for this retrospective study.

### Study population

**Fig. 1:**
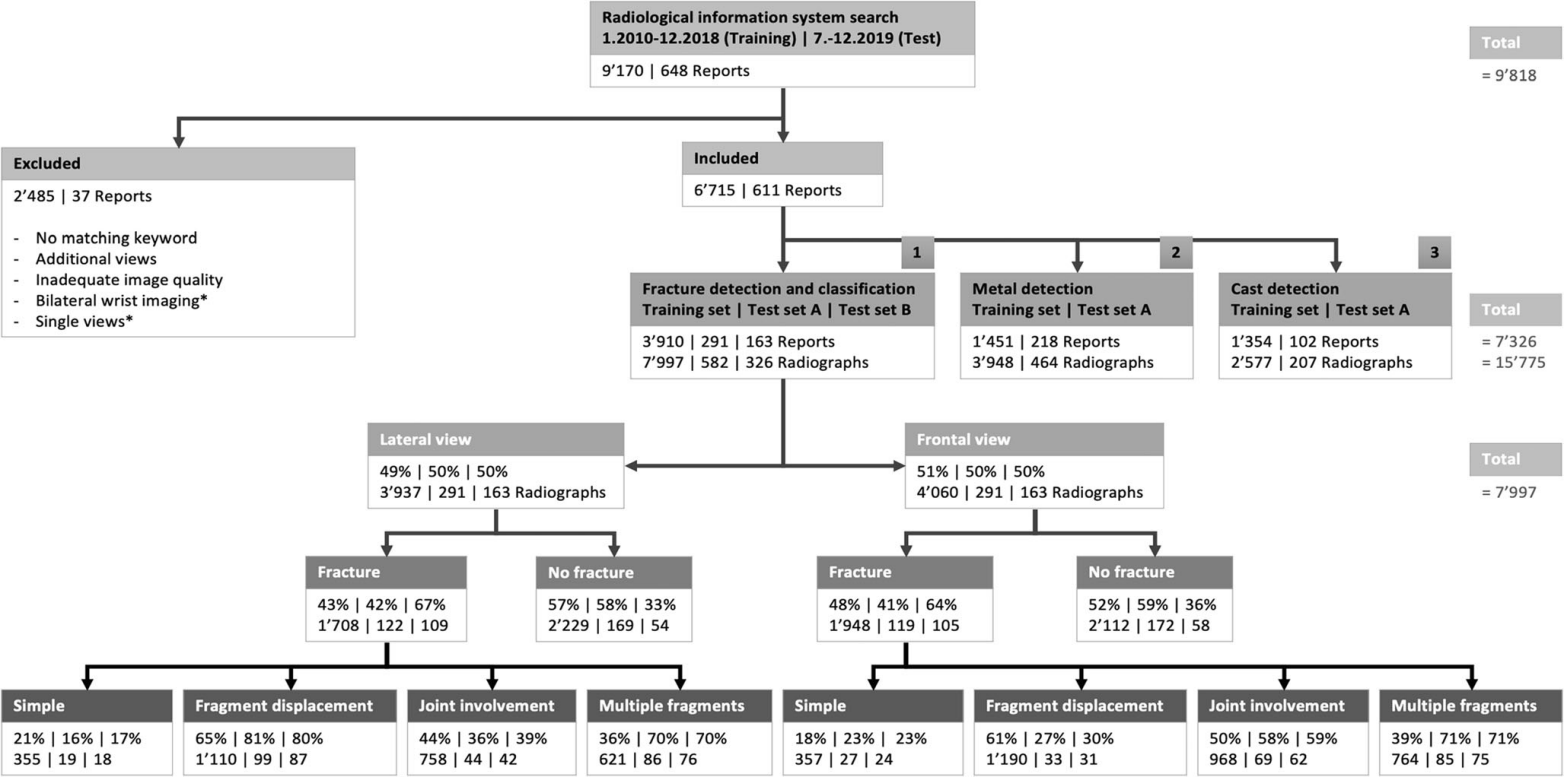
Flowchart demonstrates the selection of training and test sets for wrist radiographs. Exclusion criteria marked with an asterisk (*) are only applicable for the test sets. One radiograph was eligible for multiple fracture classification labels. Test set A was rated by two musculoskeletal radiology experts and reflects the standard of reference. Test set B is a subset of A and used to compare three radiology residents to the algorithms

A retrospective search of our radiology information system using a custom-written PACS-crawler was performed to select radiology reports of wrist radiographs between April 2010 and December 2019. They were searched for the key phrase “distal radius fracture” with a total of 9,818 detected reports (Fig. 1). These were sorted into three categories: distal radius fracture detection and classification (category 1); metal detection (category 2); cast detection (category 3). Category 1 included only reports from the emergency department and their reports with the keywords “osteosynthesis,” “plate,” “cast,” and/or “follow-up” were excluded as well as additional radiographic views, such as of the scaphoid, hand, or forearm. Studies with one single view were included in the training set, but manually excluded from the test set. Category 2 consisted of reports from all referring departments with the keywords “osteosynthesis” and/or “plate.” Category 3 included reports with the keyword “cast” from the same selection. A total of 7,326 reports with 15,775 radiographs were included.

### Training and test sets

The included reports were split into a training and a test set for each of the three investigated categories (Fig. 1). The training sets included studies from April 2010 to December 2018, and the test sets studies from July 2019 to December 2019.

Radiographs of the fracture detection and classification category were sorted by the view. For the fracture detection training set, the labels “fracture” or “no fracture” were assigned to all radiographs depending on key phrases indicating presence or absence of distal radius fracture in the report. For the fracture classification training sets, only radiographs with the label “fracture” were eligible. The classification labels were distributed according to key phrases, including “displacement,” “ulnar,” “radial,” “dorsal,” “volar/palmar,” “intraarticular,” “extraarticular,” and “multifragmented” as proposed by the AO/OTA classification [16]. Reports that did not match either of the keywords were not classified and were referred to as simple fractures.

The label “metal” was applied to radiographs in the metal category. The label “no metal” was applied to all radiographs from the fracture detection and classification category. The procedure for the cast category was equal. For both categories, the views were used indistinctively.

**Fig. 2:**
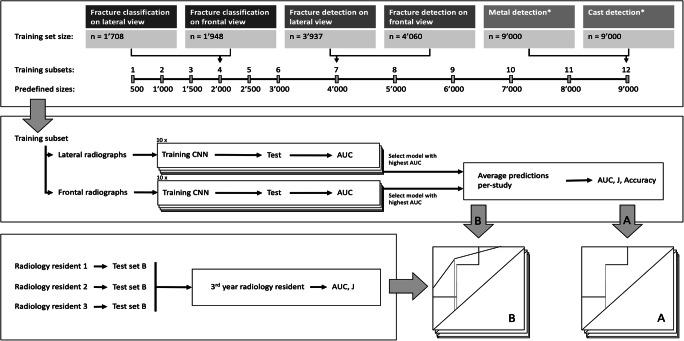
Flowchart shows the training and test architecture. Top—Set-up of training subsets and their sizes. All subsets were in accordance with the predefined sizes, except the biggest subset, which contained all radiographs available for each category. Middle—Set-up to develop artificial intelligence (AI) algorithms. Performance was evaluated with area under the receiver operating characteristics curve (AUC), Youden’s J statistic (J), and accuracy. Bottom—Set-up to determine the radiology resident`s performance and performance evaluation on test set A (AI only) and B (AI and radiology residents). *Metal and cast detection training sets were limited to 9,000 images and included both views simultaneously; therefore, the model was used directly, avoiding the splitting and averaging of predictions steps (see middle set-up). DCNN = deep convolutional neural network

To assess the suitability of labels based on key phrases extracted from radiology reports as input for DCNN training, standardized training subsets were randomly generated with predefined subset sizes ranging from 500 to a maximum of 9,000 radiographs (Fig. 2), leading to a total of 62 subsets. Only the metal and cast detection training sets contained more than 9,000 images and no improvement was expected from training set sizes beyond that number. For the training sets in the fracture detection and classification category, all available radiographs were allocated to the largest subset, which did not reach the predefined size. In each randomly generated subset, the original ratio between positive and negative labels was maintained (Fig. 1). Each subset was split into 90% training and 10% validation.

### Artificial intelligence algorithms

The selected DCNN architecture was ResNet 18, pre-trained on ImageNet using the DL framework Pytorch (version 1.2, https://pytorch.org). Trainings and tests were run on a NVIDIA GTX 2080TI (NVIDIA) with 11GB of RAM. The batch size was set to 24. An optimizer stochastic gradient descent (SGD) was used with an initial learning rate of 0.001 and a momentum of 0.9. The learning rate was reduced by 0.1 every seven epochs. Data was augmented with a horizontal flip and, with a probability of 50%, application of an affine transformation with up to five degrees and scaling between 90 and 110%. Training was performed in 15 epochs. For training and tests, the images were center cropped to a size of 1024 × 1024 pixels and resized to 224 × 224 pixels with a subsequent normalization of pixel values according to the ImageNet mean and standard deviation.

**Fig. 3:**
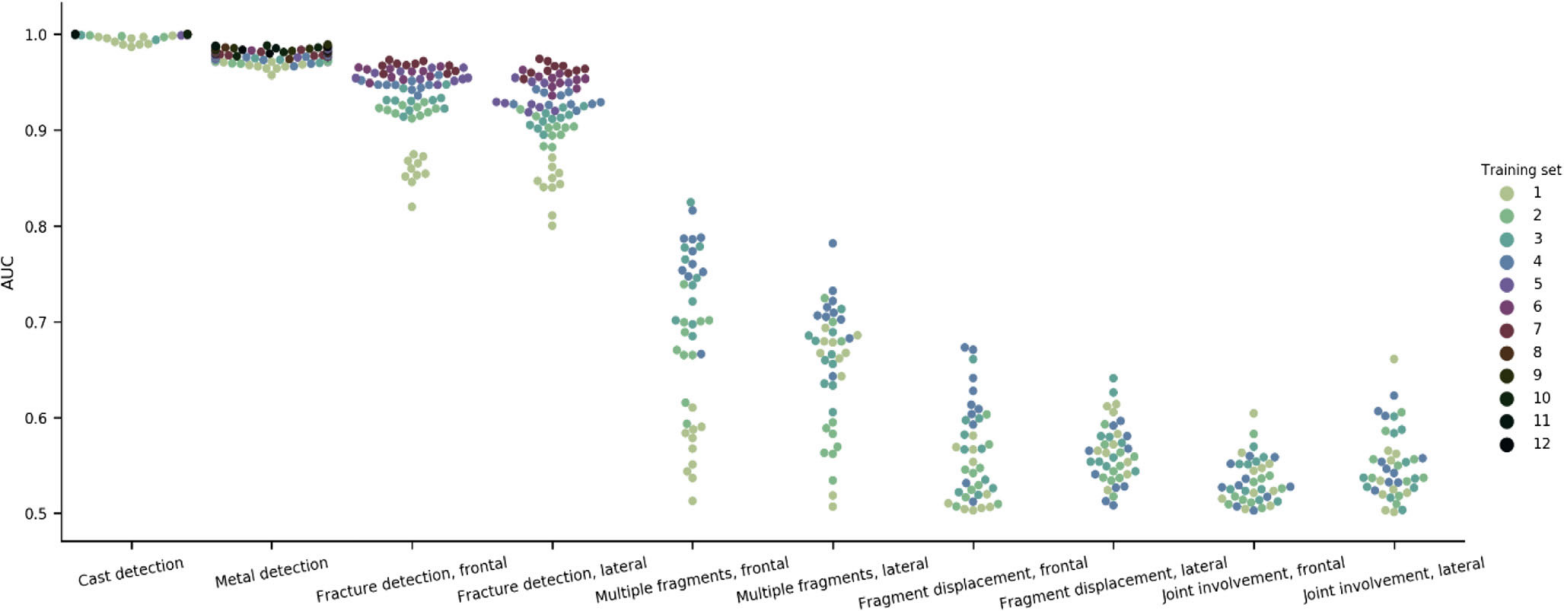
Artificial intelligence models performance for distal radius fracture detection, classification, and cast and metal detection on test set A. The performance was measured with the area under the receiver operating characteristics curve (AUC). The graph shows the effect of an incrementally increased training subset size between 500 (subset 1) and 9,000 (subset 12) radiographs on model performance, and the possible performance variation per training subset

Two models trained with one set can perform substantially different (Fig. 3). To receive a good estimate on the maximal potential of a training subset size, ten models were trained on each subset, leading to a total of 620 models.

### Test set A

For fracture detection and classification, the standard of reference was set by two musculoskeletal senior radiologists with 14 (B.K.) and 15 (A.H.) years of experience. They labeled 582 wrist radiographs in consensus and blinded to clinical information viewing them in pairs on Nora (Nora Medical Imaging Platform Project) as follows: presence or absence of distal radius fracture, fragment displacement, joint involvement, and multiple fragments. Ground truth labels were assigned to each radiograph separately. For metal and cast detection, one reader (P.T.) labeled a total of 671 wrist radiographs.

### Test set B

From the test set A, 326 radiographs were systematically selected to create test set B. Goals were to exclude metal and cast, and reduce the amount of fracture negatives. Three radiology residents (2nd year (R.S.), 3rd year (V.H.), and 4th year (F.P.)) analyzed test set B independently and blinded to clinical information on Nora with a simultaneous display of both views, as follows: presence or absence of distal radius fracture, fracture displacement, joint involvement, and multiple fragments. Radiology residents’ answers were registered for each pair of radiographs. Prior to that, all three readers received a tutorial introduction on the use of Nora and the applied criteria on wrist radiographs not included in the test set B.

### Statistical analysis

Area under the receiver operating characteristic curve (AUC) and Youden’s J statistic (J) was determined for the averaged results of the radiology residents, and for all models and algorithms (Fig. 2). To determine correlation between training subset size and model performance, Spearman‘s correlation coefficient (ρ) was used for an all-models per training subset and also for a best-model per training subset analysis. The best model was determined by AUC.

To simulate the human interdependent evaluation in fracture detection and classification on radiographs, only the models with the highest AUC in each test set were used for the respective analysis. First, predictions, which were calculated for each view separately, were averaged on a per-study base. If only one view was rated positive for a finding, this single view was assessed with the algorithm and the other view was assigned zero. This average of the two best models is referred to as algorithm. In addition to AUC and J, the accuracy on test set A was calculated.

Results of the three radiology resident readers (2nd to 4th year of residency) were averaged (Fig. 2). The wrongly classified radiographs were manually reviewed.

Chi-square test was used to compare the frequency of fractures and classes between the test set A and training set. Interobserver agreement between the standard of reference, radiology resident analysis, and AI algorithms and models was assessed using Fleiss’ kappa statistics (κ). According to Landis and Koch, a kappa value of 0–0.20 indicates slight agreement; 0.21–0.40, fair agreement; 0.41–0.60, moderate agreement; 0.61–0.80, substantial agreement; and 0.81–1, almost perfect agreement [17]. A *p* value of < 0.05 was considered statistically significant. For all analyses, Python 3 (Python Software Foundation) and SPSS 26 (IBM SPSS Statistics for Windows) were used.

## Results

### Study population

Training and test set A did not differ for the frequency of fracture presence on the lateral view (43%/42%; *p* = 0.67) and joint involvement on both views (frontal, 50%/58%; *p* = 0.09; lateral, 44%/36%; *p* = 0.09; Fig. 1). However, they differed for the presence of a fracture on the frontal view (48%/41%; *p* = 0.02), multiple fragments (frontal, 39%/71%; *p* < 0.001; lateral, 36%/70%; *p* < 0.001), and fragment displacement (frontal, 61%/27%; *p* < 0.001; lateral, 65%/81%; *p* < 0.001) on both views.

### Training subset size and AI performance

Table 1 and Fig. 3 display correlations of the training subset sizes and the performance of models measured by AUC. Regarding the total 620 models, a statistically significant positive correlation was evident on both views for fracture detection (frontal, ρ = 0.947, *p* < 0.001; lateral, ρ = 0.946, *p* < 0.001), and classification of multiple fragment (frontal, ρ = 0.856, *p* < 0.001; lateral, ρ = 0.489, *p* = 0.0013), as well as on the frontal view for fragment displacement (ρ = 0.595, *p* < 0.001). The correlation was equally traceable for detection of metal (ρ = 0.740, *p* < 0.001) and cast (ρ = 0.722, *p* < 0.001).

**Table 1:** Spearman’s correlation coefficient (ρ) between training subset size and model performance measured by area under the receiver operating characteristics curve (AUC) with two separate analyses.

		All models		Best models	
	View	ρ	*p*	ρ	*p*
Fracture	Frontal	0.947	**< 0.001**	1.000	**< 0.001**
	Lateral	0.946	**< 0.001**	0.964	**< 0.001**
Fragment displacement	Frontal	0.595	**< 0.001**	1.000	**< 0.001**
	Lateral	−0.119	0.464	0.000	1.000
Joint involvement	Frontal	0.046	0.780	−0.800	0.200
	Lateral	0.200	0.215	−0.400	0.600
Multiple fragments	Frontal	0.856	**< 0.001**	1.000	**< 0.001**
	Lateral	0.489	**0.001**	0.800	0.200
Metal	Both	0.740	**< 0.001**	0.522	0.067
Cast	Both	0.722	**< 0.001**	0.305	0.335

Best models per training subset measured AUC. A *p* value < 0.05 was considered statistically significant (indicated in bold)

The correlation was similar when calculated for only the best 62 models. Of these, all but multiple fragments classification on the lateral view (ρ = 0.800, *p*=0.2) reached statistical significance. The correlation for metal (ρ = 0.522, *p* = 0.07) and cast (ρ = 0.305, *p*=0.34) detection was lower, due to an already very good performance using small numbers (Fig. 3).

The performance of models for fracture detection, developed with training sets between 500 and 2,000 radiographs and measured by AUC, was 0.82–0.96 (frontal), and 0.80–0.94 (lateral). Only three of the classification tasks showed statistically significant correlation: multiple fragments 0.51–0.78 (lateral) and 0.51–0.82 (frontal) as well as fragment displacement 0.50–0.67 (frontal).

### Radiology resident analysis compared to AI

**Fig. 4:**
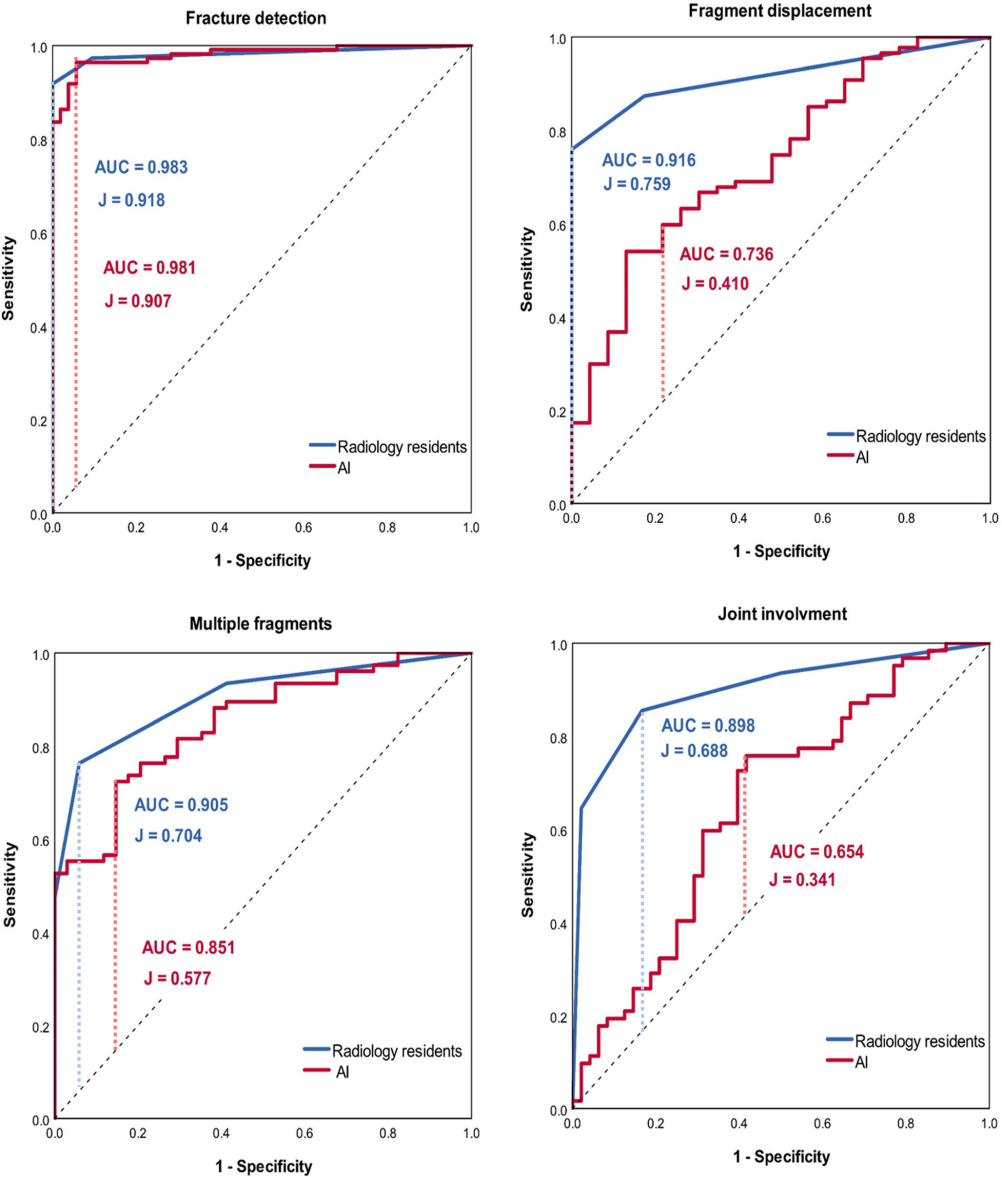
Area under the receiver operating characteristics curve (AUC) of the per-study average of the best artificial intelligence (AI) algorithms and radiology resident analysis. J = Youden’s J statistic

Table 2 and Fig. 4 depict the performance of AI compared to the radiology resident image analysis. The algorithm and radiology residents did not show a significant difference for fracture detection (AUC 0.981/0.983, J 0.907/0.918; *p* = 0.864) and classification of multiple fragments (AUC 0.851/0.905, J 0.577/0.704; *p* = 0.112). However, their performance significantly differed for classification of fragment displacement (AUC 0.736/0.916, J 0.410/0.759; *p* = 0.002) and joint involvement (AUC 0.654/0.898, J 0.341/0.688; *p* < 0.001). The residents rated nine images as false negatives. The algorithm produced three false positive and four false negative images. Two of the false positives included bony superimposition appearing as cortical irregularity and the other showed radioscaphoid osteoarthritis secondary to calcium pyrophosphate deposition with scapholunate advanced collapse. The false negatives were all minute fractures.

**Table 2:** Performance of best artificial intelligence (AI) algorithms and standard of reference (test set A) and of AI and radiology residents (test set B)

	Test set A			Test set B				
	AI			AI		Radiology residents		
	AUC	95% CI	Accuracy	AUC	95% CI	AUC	95% CI	*p*
Fracture	0.975	0.957–0.992	0.938	0.981	0.963–0.998	0.983	0.965–1.000	0.864
Fragment displacement	0.589	0.463–0.715	0.597	0.736	0.624–0.847	0.916	0.871–0.961	**0.002**
Joint involvement	0.618	0.516–0.720	0.637	0.654	0.549–0.760	0.898	0.841–0.956	**< 0.001**
Multiple fragments	0.842	0.774–0.911	0.782	0.851	0.780–0.922	0.905	0.853–0.956	0.112
Metal*	0.989	0.982–0.996	0.976					
Cast*	1.000	1.000–1.000	1.000					

Data of algorithms display per-study average analysis results. *AUC* area under the receiver operating characteristics curve, *CI* confidence interval. Test set B: A *p* value < 0.05 was considered statistically significant (indicated in bold). *Values of the best model are given, views were not considered in these categories

### Interobserver agreement

Agreement between the standard of reference, radiology resident analysis, and AI models was almost perfect for fracture detection (κ 0.83–0.88; Table 3). For fracture classification, overall agreement varied from fair to substantial (κ 0.21–0.69). Agreement between each of the three radiology residents and standard of reference was almost perfect for fracture detection (κ 0.85–0.9) and ranged from fair to moderate (κ 0.31–0.63) for fracture classification.

**Table 3:** Interobserver agreement between standard of reference, radiology residents, and per-study average of the best artificial intelligence (AI) algorithms

		Fracture detection	Fracture classification		
			Fragment displacement	Joint involvement	Multiple fragments
Standard of reference	Radiology residents	0.88	0.57	0.69	0.62
AI	Standard of reference	0.83	0.24	0.28	0.51
AI	Radiology residents	0.84	0.21	0.26	0.63
Reader 1	Reader 2	0.85	0.54	0.40	0.31
Reader 1	Reader 3	0.90	0.63	0.55	0.48
Reader 2	Reader 3	0.88	0.47	0.57	0.52
Reader 1	Standard of reference	0.87	0.61	0.51	0.60
Reader 2	Standard of reference	0.88	0.37	0.61	0.42
Reader 3	Standard of reference	0.87	0.46	0.63	0.52

Radiology residents comprise reader 1–3. Kappa values according to Landis and Koch [17]

## Discussion

This study evaluated the potential of a ResNet18 DCNN to develop models which detect cast, metal, and distal radius fractures on wrist radiographs, and classified fractures, utilizing labels based on radiology reports.

For the detection of metal and cast, the models achieved excellent AUCs of 0.99 and 1.00, respectively. Automated detection of metal from radiographs in a certain body region may be used to flag the patient’s chart and is of importance when further radiological examinations are planned, such as computed tomography or magnetic resonance imaging, to appoint a dedicated scanner with a metal artefact reduction protocol [18].

The best fracture detection algorithm (AUC 0.98, accuracy 0.94) performed similar to the radiology residents (AUC 0.98). Five studies on distal radius fracture detection using different DCNN models reported a similar performance (AUCs of 0.93–0.98) using the traditional labeling approach [4–6, 12, 13]. However, their ground truth for data labeling varied and included orthopedic surgeons [5], orthopedic specialists [6], radiology residents [13, 14], and general radiologists [4]. Lindsey et al [6] stated that only expert-given labels ensure minimal noise in the training set, and assumed that only an algorithm trained with high-quality labels can be trusted. Using manually identified key phrases on radiology reports, the models of Olczak et al [12] reached a lower accuracy of 0.83 for fracture detection on wrist, hand, and ankle radiographs. To determine their standard of reference and comparison, single radiographs cropped and resized to 256 × 256 pixels were used, which artificially restrained the humans. In this study, radiographs were viewed in a realistic setting for ground truth and comparison. Therefore, the results can be expected to be reproducible in clinical practice. The considered studies suggested superiority of labels given by musculoskeletal experts, which was not confirmed by our results. High-quality labels are not expected to significantly improve the models’ performance. The models failed primarily on different radiographs than the radiology residents do; therefore, clinical testing of the best fracture detection models is indicated.

The algorithm performance on multiple fragments classification (AUC 0.85, accuracy 0.78) reached comparable results to the radiology residents. In contrast, algorithms for fragment displacement (AUC 0.74, accuracy 0.60) and joint involvement (AUC 0.65, accuracy 0.64) performed significantly inferior to the radiology residents.

Automated fracture classification is an essential step towards automated radiology reporting and has not been available for the wrist to date. Chung et al [9] developed an algorithm to classify proximal humeral fractures on frontal radiographs cropped to the region of interest. Their classification considered only displaced fragments and the anatomical region. Their algorithms performed similar to experts and achieved better results (AUC 0.90–0.98, J 0.71–0.90) than ours (AUC 0.65–0.85, J 0.34–0.58). However, the results are not directly comparable since the AO/OTA classification evaluates joint affection and multiple fragments independent from fragment displacement. Therefore, further research on fracture classification is needed.

Models were developed on training sets in predefined and incremental sizes to better understand the potential of labels based on key phrases for pattern detection on wrist radiographs. The range of AUC achieved by models using training sets from 500 to 2’000 radiographs were used to rank the difficulty of pattern detection tasks. It was shown that automated detection of cast (AUC, 0.99–1.00), metal (0.96–0.98), and fracture (frontal, 0.82–0.96) were easily feasible. Automated classification of multiple fragments was rather difficult (frontal, 0.51–0.82; lateral, 0.51–0.78), classification of fragment displacement difficult on the frontal (0.50–0.67) and not feasible on the lateral view. Classification of joint affection was not feasible on either view. The degree of correlation between training set size and performance may be used to assess the quality of labels. We found that the interobserver agreement between residents and standard of reference was associated with the correlation between training set size and performance, if joint involvement was excluded from the comparison. The interobserver agreement in this study is in line with the literature on reproducibility of the AO/OTA classification [19, 20]. Therefore, we conclude that a keyword-based search can only generate accurate labels for items with high interobserver agreement. Joint involvement may have been inconsistently reported in our unstructured radiology reports.

Several limitations apply to this study. First, our radiology reports were written by radiology residents and radiologists with varying expertise in musculoskeletal imaging and are not structured, which may have influenced the label quality. Second, we did not use an advanced dual-input model or ensemble learning which prevented us from finding the best combination of models. As described by Pan et al [21] when building an ensemble, the best combination is obtained from models which contrast each other ideally, however may not be the best individual models and may include more than two models. Third, as we only included images of one institute, the total number of radiographs was limited after applying all exclusion criteria to 15,775 images. A bigger training set would increase the robustness of the models and alter the performance according to the observed tendencies.

The models trained on a DCNN with report-based labels to detect distal radius fractures on radiographs are suitable to aid as a secondary reading tool; models for fracture classification are not ready for clinical use. Bigger training sets lead to better models in all categories except joint affection.

## Abbreviations

AI: Artificial intelligence
AUC: Area under the receiver operating characteristic curve
CI: Confidence interval
DCNN: Deep convolutional neural networks
DL: Deep learning
J: Youden’s J statistic
SGD: Stochastic gradient descent
κ: Fleiss’ kappa statistics
ρ: Spearman‘s correlation coefficient

## Declarations

### Guarantor

The scientific guarantor of this publication is Anna Hirschmann.

### Statistics and biometry

One of the authors (Felix Amsler) has significant statistical expertise.

### Methodology

• retrospective

• diagnostic study

• performed at one institution
